# In Situ Synthesis of Carbon Nanotube–Steel Slag Composite for Pb(II) and Cu(II) Removal from Aqueous Solution

**DOI:** 10.3390/nano12071199

**Published:** 2022-04-03

**Authors:** Pengfei Yang, Fangxian Li, Beihan Wang, Yanfei Niu, Jiangxiong Wei, Qijun Yu

**Affiliations:** 1 School of Materials Science and Engineering, South China University of Technology, Guangzhou 510641, China; yang.pf@mail.scut.edu.cn (P.Y.); msbeihanwang@mail.scut.edu.cn (B.W.); jxwei@scut.edu.cn (J.W.); 2 Guangdong Low Carbon Technology and Engineering Center for Building Materials, Guangzhou 510641, China; 3 School of Civil Engineering, Guangzhou University, Guangzhou 510006, China; yanfei.n@gzhu.edu.cn

**Keywords:** carbon nanotubes, steel slag, chemical vapor deposition, heavy metals, adsorption, wastewater treatment, efficient and low cost

## Abstract

Methods and materials that effectively remove heavy metals, such as lead and copper, from wastewater are urgently needed. In this study, steel slag, a low-cost byproduct of steel manufacturing, was utilized as a substrate material for carbon nanotube (CNT) growth by chemical vapor deposition (CVD) to produce a new kind of efficient and low-cost absorbent without any pretreatment. The synthesis parameters of the developed CNT–steel slag composite (SS@CNTs) were optimized, and its adsorption capacities for Pb(II) and Cu(II) were evaluated. The results showed that the optimal growth time, synthesis temperature and acetylene flow rate were 45 min, 600 °C and 200 sccm (standard cubic centimeter per minute), respectively. The SS@CNTs composite had a high adsorption capacity with a maximum removal amount of 427.26 mg·g^−1^ for Pb(II) and 132.79 mg·g^−1^ for Cu(II). The adsorption proceeded rapidly during the first 15 min of adsorption and reached equilibrium at approximately 90 min. The adsorption processes were in accordance with the isotherms of the Langmuir model and the pseudo-second-order model, while the adsorption thermodynamics results indicated that the removal for both metals was an endothermic and spontaneous process. This study showed that compared with other adsorbent materials, the SS@CNTs composite is an efficient and low-cost adsorbent for heavy metals such as lead and copper.

## 1. Introduction

Heavy metals, such as lead (Pb), copper (Cu), cadmium (Cd), chromium (Cr) and mercury (Hg), are nonbiodegradable and highly toxic, and can accumulate in the biosphere via the food chain [1]. The accumulation of heavy metals in the human body can lead to cancer and other tissue lesions [2,3]. Compared with other heavy metals, lead and copper are not only widely used in industrial manufacturing but are also constantly exposed to people in daily life. Notably, lead and its compounds are defined as class II carcinogens by the International Agency for Research on Cancer (IARC), as they are a possible cause of lung and stomach cancers. Moreover, excessive intake of lead and copper is likely to cause serious damage to the kidney and nervous and respiratory systems [4,5,6]. Consequently, methods and materials that effectively remove lead and copper from wastewater are urgently needed.

At present, various methods are applied in the elimination of lead and copper from wastewater such as chemical precipitation, electrodialysis and membrane filtration [7,8,9,10,11]. Although these methods are effective in removing copper and lead from wastewater, they have their own limitations. The chemical precipitation method requires an additional operational cost in the removal of the hazardous residues produced by itself. The main drawback with electrodialysis is high energy consumption, while the high cost in production and the complexity of residue treatment limit the application of the membrane filtration method [12,13]. Compared to these methods mentioned above, adsorption has proved to be a better and more widely used approach because of its operational ease, high sorption capacity and safety for the environment [14,15]. A variety of absorbent materials have been used in the removal of lead and copper, including chitosan, zeolites, diatomite, activated carbon, carbonized sawdust and lignite. However, some of these adsorbents have low removal efficiency for lead and copper [13,16,17,18]. Carbon nanotubes (CNTs), a tubular carbon-based nanomaterial, have been validated as a novel and effective adsorbent for lead and copper removal owing to their large specific surface area, abundant porosity, outstanding chemical stability and the potential to be modified by functional groups [15,18,19].

Unfortunately, their hydrophobicity and high aspect ratio makes CNTs more prone to agglomeration, which leads to insufficient contact with the metal ions. Combined with their high cost, the above weaknesses have limited the application of CNTs [20]. Most of the recent research has focused on the pretreatment of CNTs to improve their dispersion and adsorption performance including surface modification and physical dispersion methods. Rodriguez et al. utilized multiwalled carbon nanotubes (MWCNTs) oxidized by nitric acid and sulfuric acid to remove Cu(II), Mn(II) and Zn(II) from water and discovered that the dispersion and adsorption efficiency of MWCNTs were augmented [21]. Oliveira et al. applied a surface active agent and ultrasonic method for the dispersion of MWCNTs, which subsequently enabled a higher removal amount of Pb(II), Cu(II), Ni(II) and Zn(II) [22].

Nevertheless, these pretreatment processes of CNTs are complex and costly, preventing their large-scale application. Fortunately, an approach whereby CNTs are synthesized on other larger adsorbent particles to form a composite by the chemical vapor deposition method (CVD) appears to reduce the agglomeration of CNTs by way of being dispersed with the dispersion of the particles in water. Moreover, this approach also provides the feasibility of large-scale adsorption application owing to the characteristics of the CVD method. As reported by other researchers, the composite seems to possess a higher adsorption capacity owing to the combination of the advantages of CNTs and other absorbents. Wang et al. synthesized CNTs on the diatomite to form a composite adsorbent for the elimination of phenol from water, and the results showed that the uptake amount of phenol by the composite significantly increased [23]. However, these materials used as the substrate for the CNTs’ growth need to be pretreated by being coated with a catalyst such as ferric nitrate, nickel nitrate or cobalt nitrate. This necessity complicates the synthesis process of the composite adsorbent, and easily causes secondary pollution due to the residual metal catalyst.

Steel slag, a low-cost byproduct of the steel industry, contains some iron-oxides (generally more than 30%, Table 1), which are regarded as an appropriate catalyst for the CNTs’ growth. Hence, steel slag can be utilized as a substrate with no need for any other pretreatment. Moreover, as reported in other studies, steel slag also has a good removal effect for Pb(II) and Cu(II). Yang et al. researched the sorption capacity of steel slag for Pb(II), Cu(II) and Cd(II) in acid solution and simultaneously evaluated the compounds formed after adsorption. The results showed that steel slag provided a remarkable removal of metal ions by ion exchange, chemical precipitation and co-precipitation to form stable compounds [24]. Shi et al. reported that the physical absorption also made a contribution to the adsorption efficiency of steel slag that was determined by the pore structure and the surface area of steel slag particles [25]. Due to the fact of its direct assemblability with CNTs and its effective adsorbability for heavy metals, steel slag can be used as a substrate for CNTs’ growth via CVD to form a new composite adsorbent. Moreover, considering waste reuse, steel slag is still recognized as a solid waste and has not been utilized well. Hence, further utilization of steel slag is an effective way to turn waste into treasure and protect the environment [26]. Therefore, this new kind of adsorbent, formed by synthesizing CNTs on steel slag, reveals a potential prospect in the application for the removal of Pb(II) and Cu(II).

In this study, CNTs–steel slag composite (SS@CNTs) was directly synthesized for the first time by means of CVD without any other pretreatment and used as a new kind of efficient and low-cost adsorbent. First, the effects of synthesis conditions, including the growth time, synthesis temperature and acetylene flow rate on the yield; crystallinity; microstructure of CNTs as well as the specific surface area and porosity characters of the composite were studied. Second, the efficiency of the adsorption of Pb(II) and Cu(II) by the synthesized SS@CNTs was evaluated and compared with other adsorbents. The adsorption process (i.e., adsorption kinetics, adsorption isotherms and adsorption thermodynamics) was investigated. Finally, the adsorption mechanism was declared in detail.

## 2. Materials and Methods

### 2.1. Materials and Reagents

The steel slag samples, used as a growth substrate for CNTs, were obtained from Baoshan Iron & Steel Co., Ltd. (Shanghai, China). X-ray fluorescence (XRF) analysis was utilized for detecting the chemical composition of the steel slag (Table 1). The steel slag contained 33.98% Fe_2_O_3_, which is generally considered to be an appropriate catalyst for the growth of CNTs [27].

The lead nitrate (Pb(NO_3_)_2_), cupric nitrate (Cu(NO_3_)_2_), sodium hydroxide (NaOH) and hydrochloric acid (HCl) were of A.R. grade and were purchased from Shanghai Macklin Biochemical Technology Co., Ltd., Shanghai, China. Deionized water was used for both the preparation of the stock solutions and the dilution of the adsorption solutions into different concentrations. The initial pH values of the solutions were adjusted with HCl (0.1 M) or NaOH (0.1 M).

### 2.2. Synthesis of the CNTs

**Figure 1:**
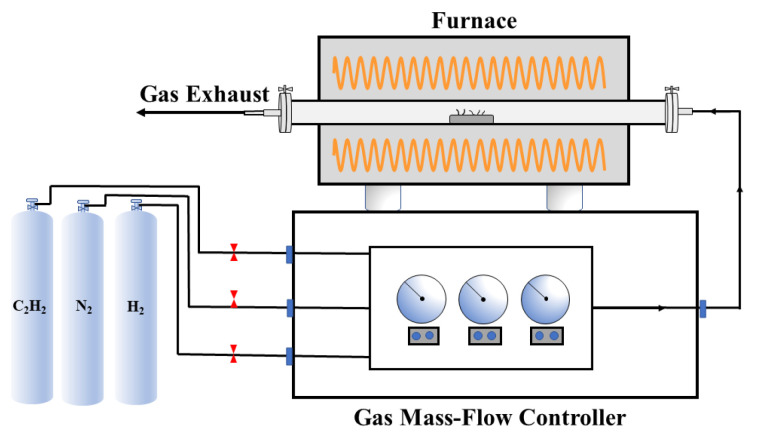
The schematic diagram of the carbon nanotubes’ synthesis device.

Figure 1 presents a diagram of the CNTs’ synthesis device, which consisted of a gas mass-flow controller and a horizontal tube furnace. The diameter of the quartz tube inside the furnace was 80 mm, and the length of the furnace heating zone was 200 mm. For synthesis of CNTs by the CVD method, acetylene, hydrogen and nitrogen were utilized as a carbon source, reducing gas and carrier gas, respectively. In this work, 10 g of well-ground steel slag was uniformly loaded into a porcelain boat and subsequently placed in the heating zone of the furnace. The whole experimental process was as follows. First, nitrogen at a flow rate of 200 sccm was injected into the furnace to remove oxygen until the quartz tube was heated to the desired synthesis temperature. Then, nitrogen, hydrogen and acetylene were introduced into the furnace at a certain rate. After a certain synthesis time, the hydrogen and acetylene flows were turned off, while nitrogen was kept at a flow rate of 800 sccm until the quartz tube had cooled down to ambient temperature. Both the rate and time varied. The yield of carbon obtained could be calculated as follows:

(1) $$\;X\left(\mathrm{wt}\%\right)=\frac{M-M^{\prime }}{M}\times 100\%$$

where X is the yield of carbon obtained (wt%); M and $M^{\prime }$ are the total mass of the synthetic product (g) and the mass of raw steel slag (g), respectively.

### 2.3. Adsorption Study

In this work, adsorption experiments were performed in a series of 200 mL polyethylene bottles with 20 mg of adsorbents and 100 mL of heavy metal ion solutions at various concentrations. The polyethylene bottles were placed in a water bath oscillator and stirred at 150 rpm (revolutions per minute) for various adsorption times. The adsorption capacity for heavy metal ions was evaluated by the following formula:

(2) $$\;q_{i}=\frac{\left(C_{i}-C_{0}\right)\times V}{m}$$

where $q_{i}$ is the adsorption capacity (mg/g); $C_{0}$ and $C_{i}$ represent the initial ion concentration (mg·L^−1^) and the ion concentration (mg·L^−1^) after equilibrium adsorption for a certain time (min), respectively; V is the volume of the solution (L); m is the amount of the SS@CNTs (g).

### 2.4. Characterization

The yield and oxidation temperature of carbon products obtained by CVD was determined by thermogravimetric analysis (TGA, Netzsch STA 449 F3 Jupiter, Selb, Germany) with a heating rate of 10 °C·min^−1^ and a temperature range of 30 to 1000 °C in an air atmosphere. The microstructural characteristics of synthetic CNTs were analyzed using a field emission scanning electron microscope (SEM, FEI NOVA NANOSEM 430, Waltham, Massachusetts, USA) at a working voltage of 10 kV. The diameters and thicknesses of the CNTs were detected by a transmission electron microscope (TEM, JEM-2100F, Tokyo, Japan). The microscopic Raman spectrometer (HJY Lab RAM Aramis, Paris, France) was employed to determine the crystallinity of CNTs with a spectra range of 400 to 4000 cm^−1^. The particle sizes of products were investigated by a laser particle analyzer (LA960S, Horiba, Kyoto, Japan). The Brunauer–Emmett–Teller (BET) method was employed to characterize the specific surface area (S_BET_) and pore characters of the products. The concentrations of heavy metal irons were tested by an inductively coupled plasma optical emission spectrometer (ICP-OES).

## 3. Results and Discussion

### 3.1. Synthesis of CNTs on Steel Slag Particles by CVD

The growth of CNTs is affected by various factors. In this study, the synthesis temperature, growth time and acetylene flow rate were varied to study the effects of synthesis parameters on the growth of CNTs utilizing steel slag as the substrate.

#### 3.1.1. Effect of Growth Time

To study the effect of growth time on the synthesis of SS@CNTs, various amounts of time were selected (i.e., 15, 30, 45 and 60 min corresponding to SS@CNTs-15, SS@CNTs-30, SS@CNTs-45 and SS@CNTs-60, respectively). The flow rates of H_2_, C_2_H_2_ and N_2_ were set as 400, 200 and 800 sccm, respectively, with a synthesis temperature of 600 °C.

**Figure 2:**
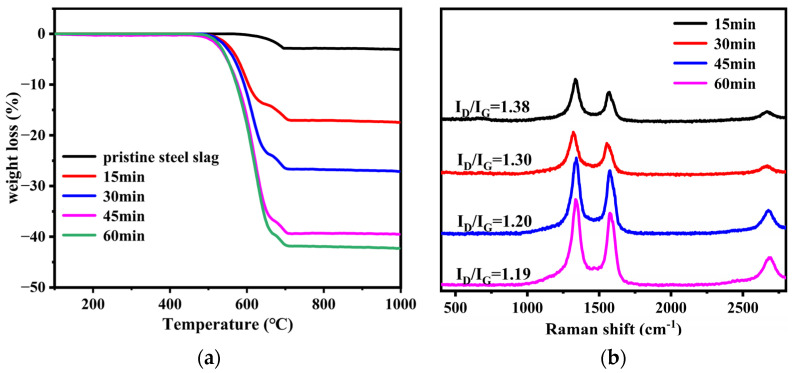
The TGA (**a**) and the Raman spectra (**b**) of SS@CNTs synthesized using different time intervals.

The results of the TG analysis are shown in Figure 2a and Table 2. The first region (460 to 670 °C) and the second region (670 to 710 °C) corresponded to the oxidization of carbon products and the decomposition of calcium carbonate (about 4 wt% loss), respectively [28,29]. The yield of carbon products on the steel slag particles increased as the growth time increased, reaching a maximum weight loss of 39% at 60 min. However, the increased rate of the yield was higher before 45 min and lower between 45 and 60 min, which was attributed to the rapid increase in precipitated carbon atoms before 45 min and the continuous decrease in active sites on the catalyst [30]. Of note, there was little difference in the initial oxidation temperature (T_0_) of the carbon products under different times, while the final oxidation temperature (T_f_) of the carbon products increased as the growth time increased. The degree of crystallization of the carbon products was basically related to the oxidation temperature. Carbonaceous species with higher crystallinity were more stable and, thus, had a higher oxidation temperature. A longer growth time ensured enough time for carbon atoms to deposit and diffuse on the substrate and then form fewer defective carbon products that had a higher oxidation temperature [31,32].

Figure 2b shows the Raman spectra of SS@CNTs samples. The D-peak in the range of 1250 to 1350 cm^−1^ corresponded to the disordered and defective character of the amorphous carbon, while the G-peak in the range of 1500–1600 cm^−1^ depended essentially on the stretching vibration of sp^2^ hybridized carbon atoms observed in most of the graphite carbonaceous species. The ratio of D-peak to G-peak intensity (I_D_/I_G_) is often used as a probe of the graphitization degree of CNTs [33]. As shown in Figure 2b, the value of I_D_/I_G_ decreased from 1.38 to 1.19 as the growth time increased from 15 to 60 min, implying that the graphitization degree of the CNTs improved as the time increased. In addition, a peak between 2600 and 3000 cm^−1^ was also related to the sp^2^ hybridized carbon atoms, and its peak intensity also had a similar trend.

**Figure 3:**
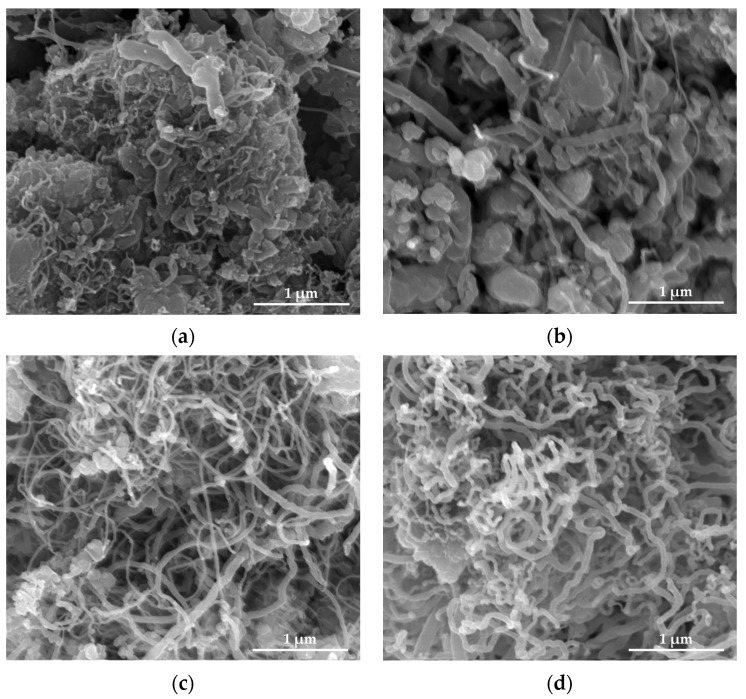
SEM images of CNTs synthesized at 15 (**a**); 30 (**b**); 45 (**c**); 60 min (**d**).

**Figure 4:**
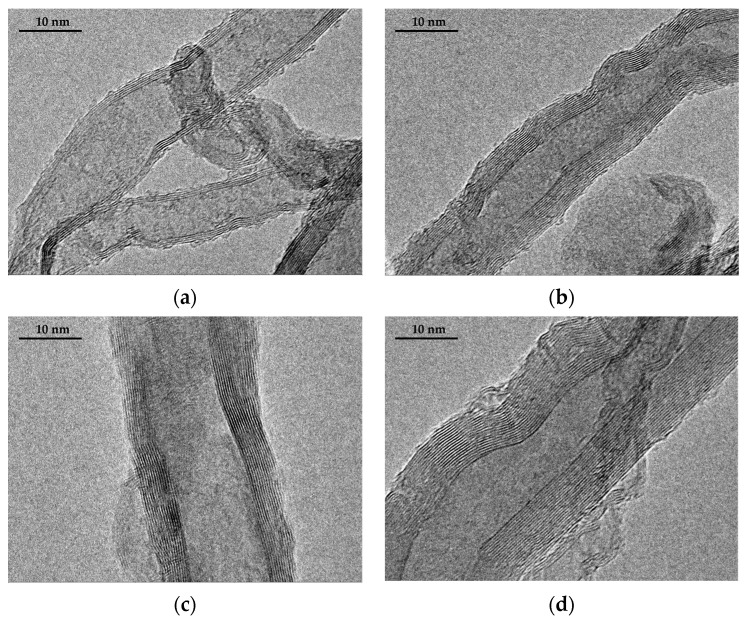
TEM images of CNTs synthesized at 15 (**a**); 30 (**b**); 45 (**c**); 60 min (**d**).

The Scanning electron microscope (SEM) images of the SS@CNTs synthesized under different times are shown in Figure 3. The CNTs were coiled around the surface of the steel slag particles, especially in the steep sections. As the growth time increased, the quantity of the CNTs and amorphous carbon increased and decreased, respectively, while the homogeneousness of the CNTs improved as the synthesis time increased. Notably, the highest length–diameter ratio of CNTs appeared at the synthesis time of 45 min. Furthermore, the CNTs that synthesized for a longer time seemed to be larger, which was clearly observed in TEM images (Figure 4). The diameters of the SS@CNTs samples were 10.3, 12.8, 17.5 and 21.3 nm, respectively, while the thicknesses were 1.6, 3.4, 4.2 and 6.2 nm, respectively. A longer growth time meant enough time for the deposition and diffusion of carbon atoms onto the particles, which increased the quantity, diameter, thickness and homogeneousness of the CNTs. Similar to the TG analysis, amorphous carbon existed in all of the samples, which was attributed to the heterogeneous and complex surfaces of the steel slag particles.

**Figure 5:**
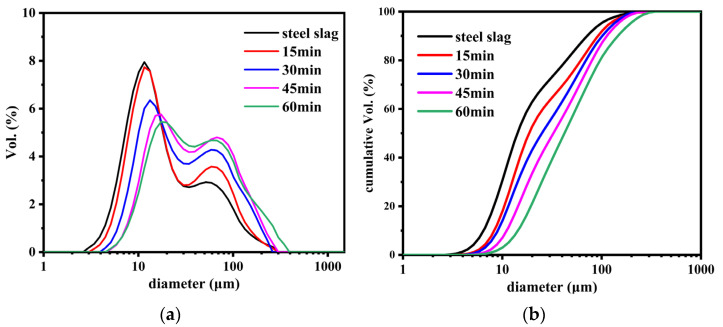
The particle size distribution (**a**) and cumulative size distribution (**b**) of SS@CNTs synthesized using different time intervals.

Figure 5 shows the particle size distribution and cumulative size distribution of SS@CNTs under different growth times. As shown in Figure 5a, the sizes of SS@CNT particles were in the range of 5–220 μm. When the growth time increased, the particle size distribution curves moved in parallel to the direction of larger particles. Similarly, from the cumulative distribution curves (Figure 5b), we found that the particle diameter corresponding to a 90% cumulative volume (D90) of SS@CNTs synthesized at 15, 30, 45 and 60 min were 71, 91, 101, 112 and 145 μm, respectively, indicating that a longer growth time produced larger particles. The BET surface area (S_BET_) and porosity characters are shown in Table 3. The S_BET_ increased before 45 min and then decreased, while the total pore volume (V_t_) and average pore diameter (D_A_) increased as the time increased. The highest value of the BET surface area was obtained at 45 min with 49.85 m^2^·g^−1^, indicating that more CNTs and other carbon products with a large surface area existed at a growth time of 45 min. The increasing trend of the V_t_ and D_A_ was related to the diameter of CNTs and the quantity of the amorphous carbon [34,35]. Based on all of the results above, an appropriate growth time was 45 min.

#### 3.1.2. Effect of Synthesis Temperature

**Figure 6:**
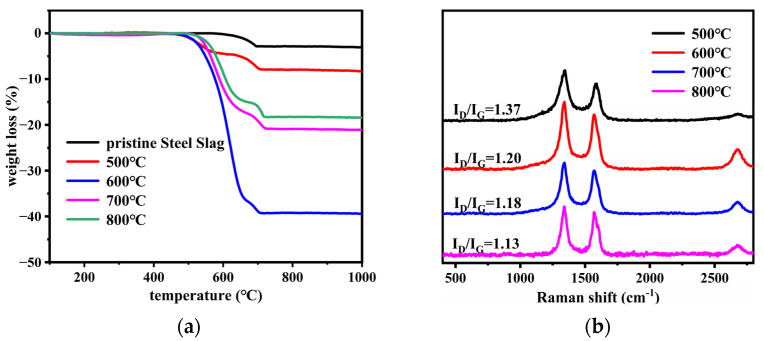
The TGA (**a**) and the Raman spectra (**b**) of SS@CNTs synthesized at various temperatures.

Figure 6a and Table 4 present the TG analysis of SS@CNTs synthesized at various temperatures (500, 600, 700 and 800 °C corresponding with samples SS@CNT-500, SS@CNT-600, SS@CNT-700 and SS@CNT-800, respectively). Furthermore, the flow rates of H_2_, C_2_H_2_ and N_2_ were 400, 200 and 800 sccm, respectively, with a synthesis time of 45 min. According to the result, the yield of CNTs on steel slag particles increased first and then decreased. It is well known that the yield of CNTs is related to the growth rate, which can be explained by the Arrhenius equation [36]. A higher temperature promotes the decomposition of the carbon precursor, the diffusion and precipitation of carbon atoms and the catalyst activity, resulting in the improvement in the growth rate. The maximum yield of 36% was at 600 °C, indicating that this is a suitable temperature for CNTs to form onto steel slag particles. Moreover, according to Table 4, as the synthesis temperature increased, the initial and final oxidation temperatures significantly increased. This phenomenon can be explained by the decrease in the amorphous carbon and the increase in the crystallinity of carbon products at 600 °C and above.

The Raman spectra of SS@CNT samples are shown in Figure 6b. The values of I_D_/I_G_ decreased from 1.37 to 1.13 as the synthesis temperature increased from 500 to 800 °C. When the synthesis temperature was below 600 °C, more amorphous carbon formed owing to the low catalyst activity and inefficient decomposition of acetylene. However, as the temperature increased to 800 °C, the value of I_D_/I_G_ decreased to 1.13, implying that the crystallinity of carbon products improved.

**Figure 7:**
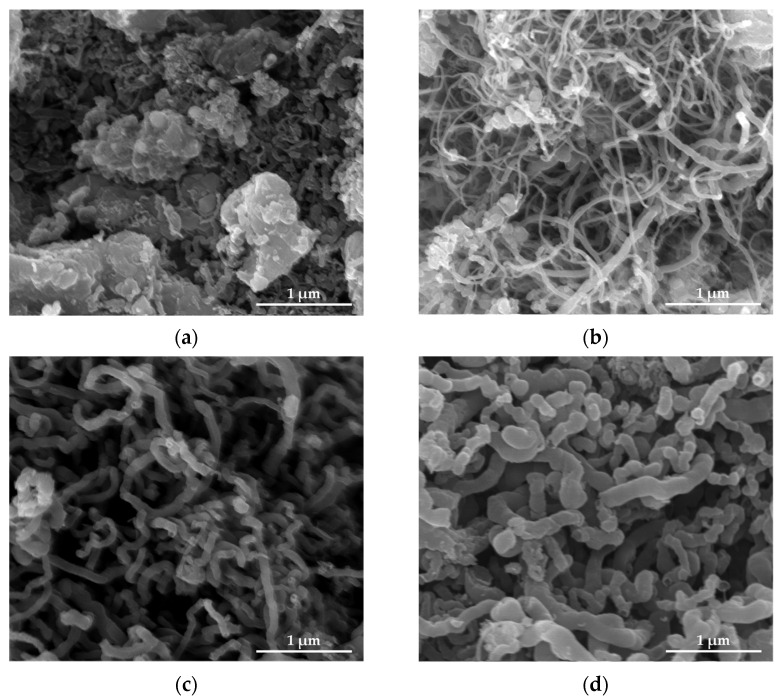
SEM images of CNTs synthesized at 500 (**a**); 600 (**b**); 700 (**c**); 800 °C (**d**).

**Figure 8:**
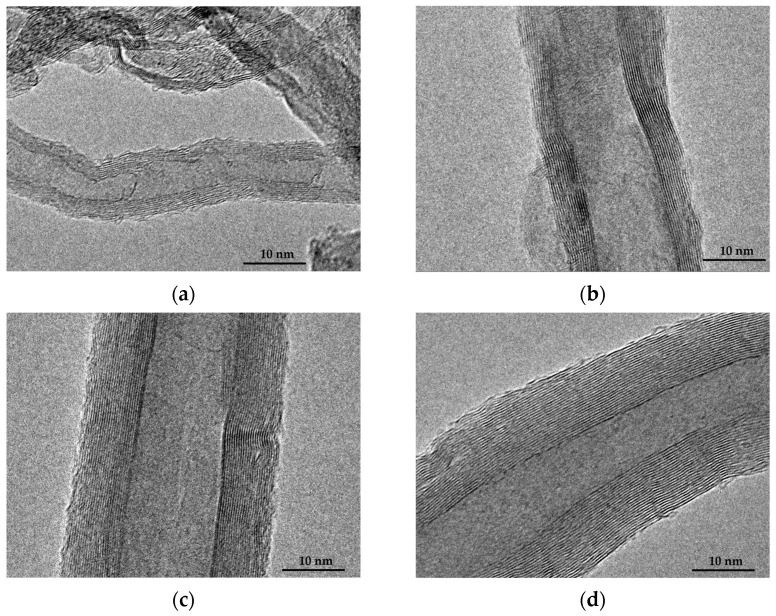
TEM images of CNTs synthesized at 500 (**a**); 600 (**b**); 700 (**c**); 800 °C (**d**).

Figure 7 shows the SEM images of SS@CNT samples. As the synthesis temperature increased, the length and quantity of CNTs increased first and then decreased, reaching the maximum at 600 °C, while the diameter of CNTs increased as the temperature increased. When the temperature was below 600 °C, fewer CNTs formed on the particles. Notably, as the temperature increased to 600 °C, the length and quantity of CNTs increased rapidly, and the highest aspect ratio of CNTs was observed. However, as the temperature continuously increased from 600 to 800 °C, the length of CNTs decreased, but the diameter increased, leading to the formation of thick and short CNTs. From the TEM result (Figure 8), we found that the diameter and wall thickness increased continually. The diameters of the samples were approximately 8.4, 17.5, 22.6 and 31.4 nm. The thicknesses of samples were approximately 2.2, 4.2, 6.5 and 6.7 nm, respectively. For a lower temperature, the activity of the catalyst as well as the diffusion and deposition rate of carbon were lower. As a result, carbon atoms were preferentially deposited on smaller particles with a higher catalytic activity to form thinner carbon nanotubes. As the temperature increased, the activity of catalyst particles increased, allowing the decomposition of carbon atoms on a larger surface of the catalyst particles, combined with the increased diffusion rate of carbon atoms on the substrate, which led to the formation of larger CNTs. An overly high temperature caused the catalyst to be inactive as a result of being covered by carbon layers, either graphitic or amorphous. Hence, shorter and thicker CNTs formed at a temperature above 600 °C [37].

**Figure 9:**
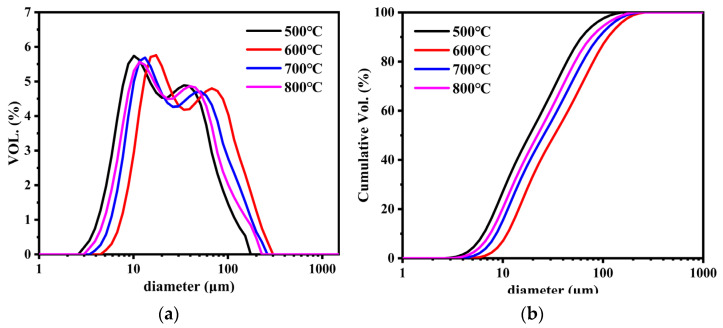
The particle size distribution (**a**) and cumulative size distribution (**b**) of SS@CNTs synthesized at various temperatures.

The particle size distribution of the SS@CNTs are shown in Figure 9. With the increase in temperature, the particle sizes increased first and then decreased, and the values of D90 under different temperatures were 62, 112, 91 and 76 μm, respectively. This phenomenon can be attributed to the variation trend in the quantity and morphology of CNTs. Table 5 presents the BET surface area and porosity characteristics of the SS@CNTs samples. As the temperature increased, the maximum BET surface area and total pore volume were obtained at a growth temperature of 600 °C, indicating that the quantities of CNTs and other carbon products with a large surface area formed. Based on all of the results above, an appropriate synthesis temperature was at 600 °C.

#### 3.1.3. Effect of the Acetylene Flow Rate

To investigate the effect of acetylene flow rate on the synthesis of SS@CNTs, several samples were prepared (sample SS@CNT-100, SS@CNT-200 and SS@CNT-300 corresponded with SS@CNTs synthetized under $V_{C_{2}H_{2}}$:$V_{N_{2}}$ = 100:800, $V_{C_{2}H_{2}}$:$V_{N_{2}}$ = 200:800 and $V_{C_{2}H_{2}}$:$V_{N_{2}}$ = 300:800, respectively) at 600 °C for 45 min.

**Figure 10:**
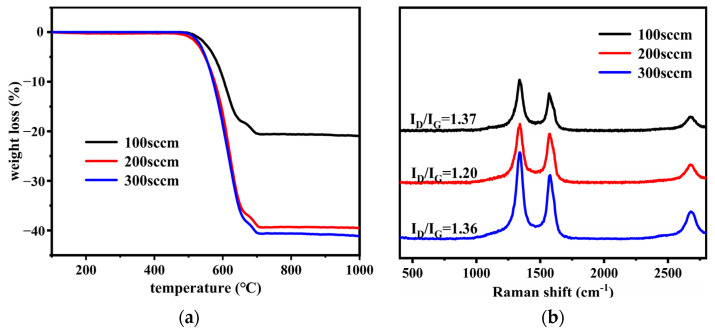
The TGA (**a**) and the Raman spectra (**b**) of SS@CNTs synthesized under various C_2_H_2_ flow rates.

Figure 10a and Table 6 show the TGA result of SS@CNTs under different C_2_H_2_ flow rates. According to the results, as the C_2_H_2_ flow rate increased, the yield of carbon products increased. When the flow rate of C_2_H_2_ increased from 100 to 200 sccm, the yield of carbon products significantly increased, from 17 to 36 wt%. A high ratio of the carbon precursor flow rate to carrier gas flow rate provided sufficient contact time between the carbon precursor and catalyst particle and, hence, formed more carbon products. From Table 6, we can see that the samples were similar among the initial oxidation temperatures, while the final temperature increased as the flow rate of C_2_H_2_ increased. This indicated that carbon products with low crystallinity existed in all samples and a high acetylene flow rate seemed to improve the crystallinity.

The Raman spectra of the SS@CNTs samples is shown in Figure 10b. As the C_2_H_2_ flow rate increased, the value of I_D_/I_G_ decreased first and then increased. The values of I_D_/I_G_ in SS@CNTs-100 and SS@CNTs-300 were higher than those in SS@CNTs-200, suggesting that the quantity of amorphous carbonaceous species and the defects of CNTs were lower under a C_2_H_2_ flow rate of 200 sccm. The crystallinity of carbon products decreased either above or below this flow rate.

**Figure 11:**
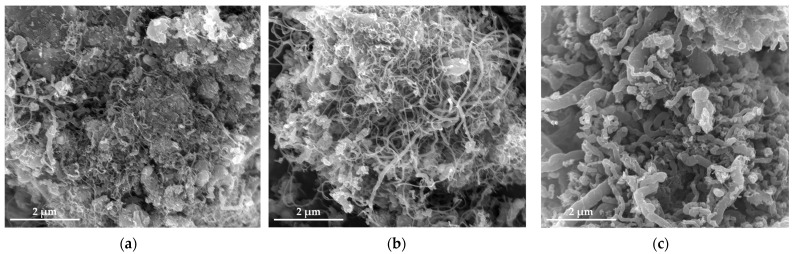
SEM images of CNTs synthesized under a C_2_H_2_ flow rate of 100 (**a**); 200 (**b**); 300 sccm (**c**).

**Figure 12:**
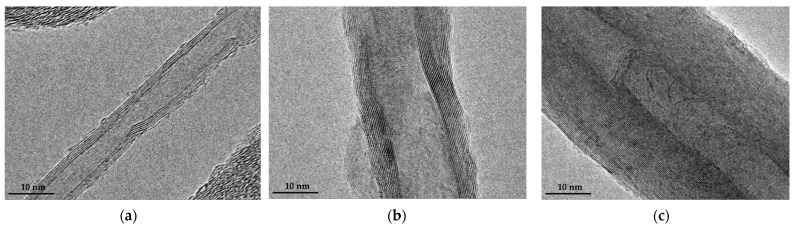
SEM images of CNTs synthesized under a C_2_H_2_ flow rate of 100 (**a**); 200 (**b**); 300 sccm (**c**).

As shown in the SEM results (Figure 11), narrow and short CNTs presented in the SS@CNTs-100, while more amorphous carbon and short and thick CNTs existed in SS@CNTs-300. In contrast, quantities of long and uniform CNTs with a high aspect ratio presented in SS@CNTs-200. From the TEM images (Figure 12), we found that the diameter and wall thickness of CNTs increased as the C_2_H_2_ flow rate increased. The diameters of CNTs in SS@CNTS-100, SS@CNTs-200 and SS@CNTs-300 were approximately 7, 13, and 41 nm, respectively. The wall thicknesses were approximately 1.2, 2.2 and 14.5 nm, respectively. As other researchers pointed out, the low flow rate of C_2_H_2_ caused a short contact time between the carbon precursor and catalyst particles; thus, the decomposition of C_2_H_2_ and the diffusion of carbon atoms were insufficient for the further growth of CNTs. However, the overly high C_2_H_2_ flow rate meant an excess carbon supply, which led to the increase in amorphous carbonaceous products and other impurities on the catalyst particles as well as on the surface of grown CNTs. Furthermore, the catalyst particles were poisoned by the amorphous carbon and, thus, formed short and thick CNTs [38,39].

**Figure 13:**
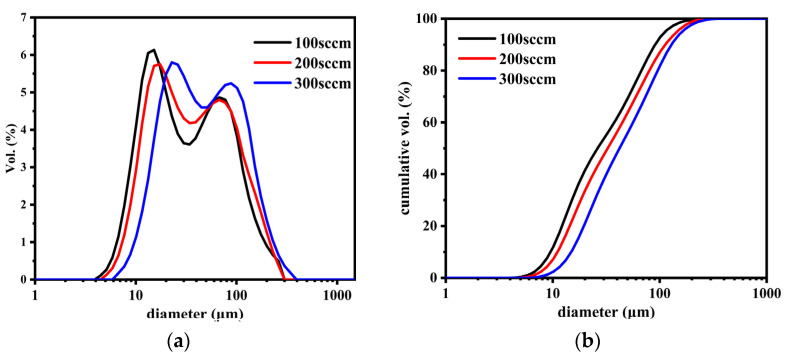
The particle size distribution (**a**) and cumulative size distribution (**b**) of SS@CNTs synthesized under different C_2_H_2_ flow rates.

Figure 13 shows the particle size distribution and the cumulative size distribution of the samples. The particle size increased as the C_2_H_2_ flow rate increased. The values of D90 in samples SS@CNTs-100, SS@CNTs-200 and SS@CNTs-300 were approximately 91, 113 and 129 μm, respectively. It seemed that the particle size was related to the quantity of carbon products on the steel slag particles. More carbon products led to larger SS@CNT particles. According to the BET surface areas and porosity characters of CNTs (Table 7), as the C_2_H_2_ flow rate increased, the BET surface area increased first and then decreased, reaching the maximum value in the SS@CNT-200 sample. In addition, as the C_2_H_2_ flow rate increased, the total pore volume increased, while the average pore diameter decreased first and then increased. As observed in the SEM images, a larger diameter in CNTs meant a larger average pore diameter in the samples. The increase in the total pore volume was the result of the increase in the entire carbon product quantity. Based on all of the results above, we concluded that 200 sccm was an appropriate C_2_H_2_ flow rate in this study.

### 3.2. Adsorption Performance

#### 3.2.1. Adsorption Kinetics

**Figure 14:**
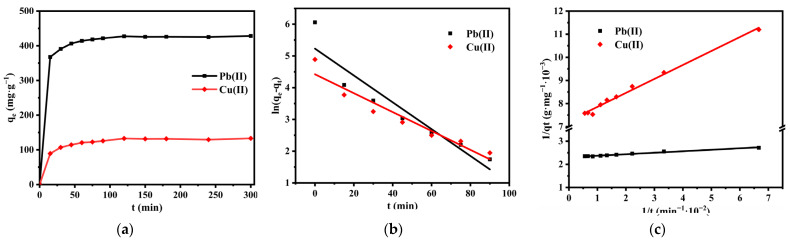
Effect of contact time on the removal of Pb(II) and Cu(II) (**a**); pseudo-first-order kinetics model (**b**); pseudo-second-order model (**c**).

A series of experiments were performed at 25 °C with an initial solution pH of 6.5. The contact time was in a range from 15 to 300 min (Figure 14a). The adsorption rate was significantly high during the first 15 min, and then gradually slowed down, reaching equilibrium at approximately 90 min. The first rapid adsorption stage suggested that there were abundant adsorption sites on the adsorbent surface, which reacted with heavy metal ions quickly. The maximum equilibrium adsorption capacities of SS@CNTs to Pb(II) and Cu(II) were 427.26 mg·g^−1^ and 132.79 mg·g^−1^, respectively. For further study of the adsorption process, the pseudo-first-order kinetic and the pseudo-second-order kinetic were carried out, which can be described by the formulas:

(3) $$\;\ln\left(q_{e}-q_{t}\right)=\ln q_{e}-k_{a}t$$

(4) $$\;\frac{t}{q_{t}}=\frac{1}{q_{e}{}^{2}k_{b}}+\frac{t}{q_{e}}$$

where $q_{e}$ is the equilibrium adsorption capacity for heavy metal ions (mg·g^−1^); $q_{t}$ is the adsorption capacity (mg·g^−1^) at time (*t*, min); $k_{a}$ and $k_{b}$ are the pseudo-first-order kinetic and the pseudo-second-order kinetic reaction rate constants, respectively.

The linear fitting diagrams and the corresponding parameters of the kinetic models are shown in Figure 14b,c and Table 8, respectively. The best fitting model was determined by comparing the correlation coefficients (*R*^2^) and the differences between the $q_{e}$ values and the experimental ones. Based on the *R*^2^ values, the pseudo-second-order kinetic model described the adsorption process more accurately than the pseudo-first-order kinetic; moreover, the values of $q_{e}$, calculated from the pseudo-second-order kinetic model, were also approximately the same as those obtained by experiments. The agreement of the pseudo-second-order process with the experimental results implied that the removal process was a chemical adsorption [40,41].

#### 3.2.2. Adsorption Isotherms

Adsorption isotherms are of great help for adsorption studies. In this study, two models (i.e., Langmuir and Freundlich) were used to fit the data obtained from adsorption equilibrium experiments, which were performed at temperatures of 15, 25 and 45 °C for 3 h. The initial concentrations of Pb(II) and Cu(II) were in a range from 80 to 160 mg·L^−1^ and 40 to 160 mg·L^−1^, respectively.

The Langmuir adsorption isotherm equation is:

(5) $$q_{e}=\frac{q_{m}\times K_{L}\times C_{e}}{1+K_{L}\times C_{e}}\;\mathrm{or}\;\frac{1}{q_{e}}=\frac{1}{q_{m}\times K_{L}\times C_{e}}+\frac{1}{q_{m}}$$

where $q_{m}$ is the maximum adsorption capacity of heavy metal ions by the adsorbent (mg·g^−1^); $K_{L}$ is the Langmuir equilibrium constant, which is concerned with the bonding stability between adsorbents and adsorbates (L·mg^−1^); $C_{e}$ is the equilibrium concentration [42].

The Freundlich adsorption isotherm equation is:

(6) $$\;q_{e}=K_{F}\times C_{e}{}^{\frac{1}{n}}\;\mathrm{or}\;\ln q_{e}=\ln K_{F}+\frac{\ln C_{e}}{n}$$

where $K_{F}$ is the Freundlich constant, and n is a constant determined by the adsorption intensity of the adsorbent [43].

**Figure 15:**
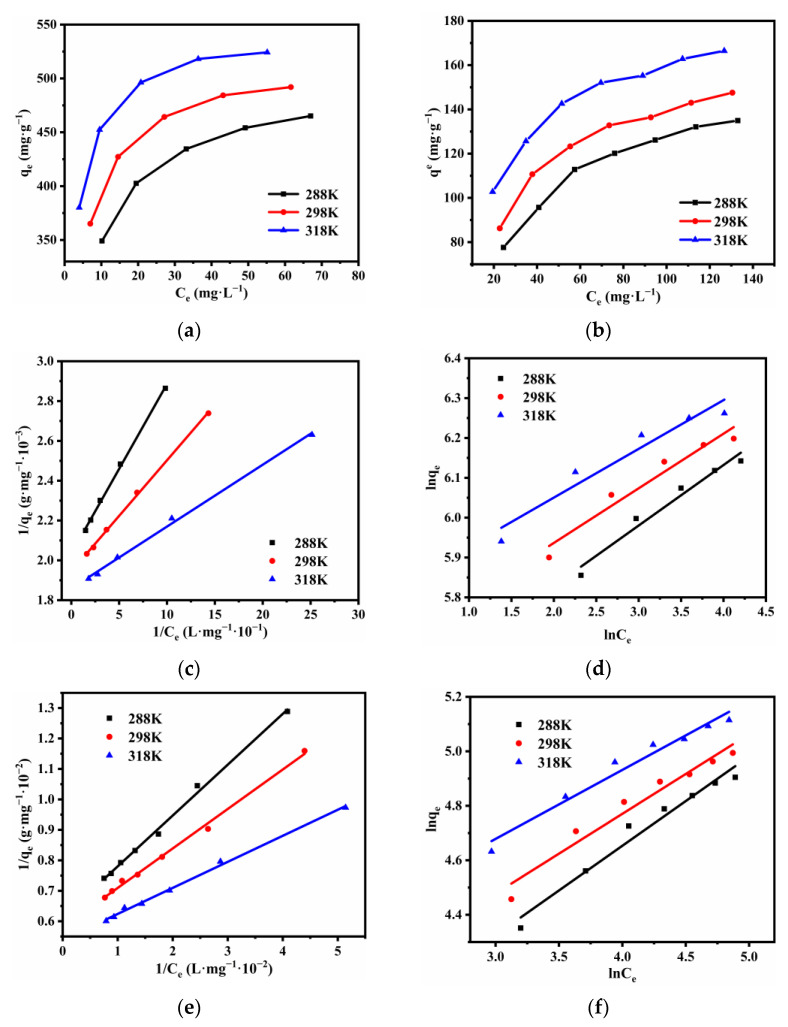
The equilibrium adsorption of Pb(II) (**a**) and Cu(II) (**b**); the Langmuir isotherm model fitting for the Pb(II) (**c**) and Cu(II) (**e**) removal process; the Freundlich isotherm model fitting for the Pb(II) (**d**) and Cu(II) (**f**) removal process.

The isothermal adsorption curves of Pb(II) and Cu(II) at the temperature of 15, 25 and 45 °C were shown in Figure 15a,b. The linear fitting charts are shown in Figure 15c–f. The corresponding parameters (i.e., $q_{m}$, $K_{L}$, $R_{L}$, $K_{F}$ and n) and the values of *R*^2^ were obtained by regressive computation of the linearized Langmuir and Freundlich isotherm models, which are presented in Table 9. From them, it is clear that Pb(II) and Cu(II) were well adsorbed by SS@CNTs. By comparing values of *R*^2^, the Langmuir model (*R*^2^ > 0.99) explained the removal of Pb(II) and Cu(II) by SS@CNTs better than the Freundlich model, implying the uniformly distributed adsorption sites as well as the monolayer adsorption of heavy metal ions on the surface of SS@CNTs. The maximum adsorption amounts calculated from the linearized Langmuir isotherm increased with the increasing temperature, which was consistent with the experimental results. In addition, all of the values of $R_{L}$ were in the range of 0 to 1, indicating the favorable adsorption of Pb(II) and Cu(II) onto SS@CNTs. Based on the parameters of the Freundlich model, the values of $k_{F}$ of both Pb(II) and Cu(II) increased as the temperature increased, suggesting that the uptake of these two ions was heightened at a high temperature. Furthermore, the values of n varied from 6.588 to 8.178 for Pb(II) and from 3.047 to 3.949 for Cu(II), which indicates that the removal of both ions occurred due to the fact of adsorption on the heterogeneous surface [44,45].

#### 3.2.3. Adsorption Thermodynamics

**Figure 16:**
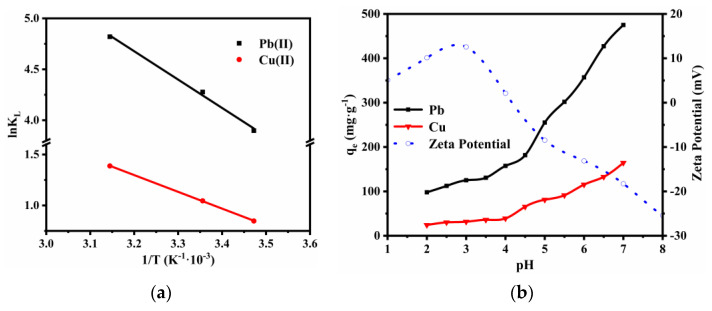
The linear regression of the curve of ln*K_L_* versus 1/*T* (**a**) and the adsorption capacity of SS@CNTs for Pb(II) and Cu(II) with pH from 2 to 7 and the values of the zeta potential at different pH values (**b**).

To investigate the thermodynamic characteristics of the adsorption process, a batch of experiments were performed at the temperatures of 15, 25 and 45 °C for 3 h with an initial pH of 6.5. The thermodynamic parameters, such as free energy ($\Delta G^{0}$), enthalpy ($\Delta H^{0}$) and entropy ($\Delta S^{0}$), were defined as follows:

(7) $$\;\Delta G^{0}=-\mathrm{RTln}K_{L}$$

(8) $$\ln K_{L}=-\frac{\Delta H^{0}}{R}\times \frac{1}{T}+\frac{\Delta S^{0}}{R}$$

where $K_{L}$ (L·mol^−1^) and R (8.314 J·mol^−1^·K^−1^) are the Langmuir equilibrium constant and the gas constant, respectively. The $\Delta H^{0}$ and $\Delta S^{0}$ were obtained from the slope and intercept of the linear plot ($\ln K_{L}$ versus $\frac{1}{T}$), which are shown in Figure 16a.

Table 10 shows the thermodynamics parameters of the adsorption process at different temperatures. The values of $\Delta G^{0}$ were negative and decreased as the temperature increased, suggesting that the adsorption of heavy metals onto SS@CNTs was spontaneous, and a high temperature was more favorable for the adsorption reaction. Moreover, the positive values of $\Delta S^{0}$ also imply a spontaneous adsorption as well as the random character of the solution–adsorbate interface. From the positive values of $\Delta H^{0}$, it can be concluded that the adsorption processes were endothermic; thus, the adsorption reaction was more likely to occur at a high temperature.

#### 3.2.4. Effect of Initial pH

The pH value affects not only the chemical state of the adsorbate ions but also ionization of functional groups on the adsorbent surface. To explore the influence of pH on the elimination of Pb(II) and Cu(II), a batch of experiments were performed under a pH range of 2 to 7 at 25 °C for 3 h. The results are shown in Figure 16b. Obviously, the uptake of these two metal ions by SS@CNTs increased as the pH values increased. Increasing pH leads to ionization of functional groups on the graphite surface that consequently encourages the interaction of metal cations with these appeared anions grafted to the surface. Moreover, the decrease in hydrogen ions competing with heavy metal ions also makes a contribution to the increase in heavy metal removal. Note that the adsorption rate increased rapidly after the pH value reached 4.5, which is related to the point of zero charge (pH_PZC_) of the absorbent surface [46]. Based on the change of the zeta potential of the SS@CNTs with the pH varying from 1 to 8, the pH_PZC_ of the absorbent surface was estimated at approximately 4.2. When the pH value was higher than pH_PZC_, the adsorbent surface was negatively charged so as to increase the likelihood of attracting positively charged heavy metal ions [2].

#### 3.2.5. Comparison and Adsorption Mechanism

By comparison of the uptake of lead and copper ions by different materials reported in the literature, which is shown in Table 11, the elimination of Pb(II) and Cu(II) by SS@CNTs was highly efficient. The adsorption, ion exchange, surface coordination and precipitation presented the main adsorption types of the elimination of Pb(II) and Cu(II) by SS@CNTs. During the adsorption process, in terms of steel slag, the calcium silicate and the free calcium oxide (f-CaO) continuously reacted with water to form calcium silicate hydrate (C–S–H) and calcium hydroxide (Ca(OH)_2_), respectively, and then released calcium ions (Ca^2+^) and hydroxide ions (OH^-^) [47]. The C–S–H was of great help in the removal of metal ions because of its high specific surface area as well as the ion exchange reaction between heavy metal ions and Ca^2+^. The OH^-^ release not only increased the pH of the solution but also formed hydroxide precipitation with heavy metal ions, which further improved the adsorption. Moreover, the hydroxyl group on the surface of the particles formed negatively charged adsorption sites that attracted the metal ions onto the surface of SS@CNTs by electrostatic interaction. The corresponding reaction formulas are as follows [24,25,48,49]:

(9) $$\;3CaO\cdot SiO_{2}+nH_{2}O\to xCaO\cdot SiO_{2}\cdot yH_{2}O+\left(3-x\right)Ca{\left(OH\right)}_{2}$$

(10) $$\;2CaO\cdot SiO_{2}+nH_{2}O\to xCaO\cdot SiO_{2}\cdot yH_{2}O+\left(2-x\right)Ca{\left(OH\right)}_{2}\;$$

(11) $$\;CaO+H_{2}O\to Ca{\left(OH\right)}_{2}\to Ca^{2+}+2OH^{-}\;$$

(12) $$\;xCaO\cdot SiO_{2}\cdot yH_{2}O+M^{2+}\to MO\cdot \left(x-1\right)CaO\cdot SiO_{2}\cdot yH_{2}O+Ca^{2+}\;$$

(13) $$2OH^{-}+M^{2+}\to M{\left(OH\right)}_{2}\;$$

The CNTs had large specific surface areas and did well in the adsorption of heavy metals. The main type of adsorption was physical absorption, such as electrostatic interaction, which has been discussed by many other researchers [50,51]. The combination of CNTs and steel slag increased the overall specific surface area, the quantity of adsorption sites and the types of adsorption, which greatly enhanced the adsorption capacity for heavy metal ions. Considering the low cost and significant adsorption capacity, the SS@CNTs’ composite adsorbent has good application prospect in the elimination of heavy metals, such as lead and copper, from wastewater.

**Table 11:** Comparison of the uptake of lead and copper ions by various adsorbents.

Heavy Metal	Adsorbent Material	Adsorbent Dosage (mg·L^−1^)	Adsorption Capacity (mg·g^−1^)	pH	Reference
Pb(II)	Acidified MWCNTs	500	166	9	[52]
	β-Cyclodextrin modified magnetic GO	200	279.21	7	[53]
	Thiol-functionalized multiwalled carbon nanotubes	200	72.4	5.5	[54]
	Silica-coated magnetic nanocomposites	4000	14.9	6	[55]
	Acid-treated multiwalled carbon nanotubes	500	97.08	5	[19]
	Nano silica spheres	1000	262	5	[56]
	Steel slag	250	53.2	6.5	This work
	Saccharomyces cerevisiae	250	238	6.5	[57]
	SS@CNTs	200	427.26	6.5	This work
Cu(II)	Acid-treated CNTs	1000	82.64	6.5	[58]
	Bacillus	2000	75.3	7	[59]
	β-cyclodextrin-modified magnetic GO	200	51.29	7	[53]
	Acid-treated multiwalled carbon nanotubes	500	24.49	5	[19]
	Double-oxidized multiwalled carbon nanotubes	667	14	4.2	[21]
	Steel slag	250	21.33	6.5	This work
	SS@CNTs	200	132.79	6.5	This work

## 4. Conclusions

In this study, an efficient and low-cost CNT–steel slag composite adsorbent was directly synthesized by means of the CVD method and applied to remove lead and copper from an aqueous solution. In this way, the CNTs on steel slag particles were evenly dispersed in the aqueous solution with the dispersion of the particles so as to obtain better contact with the metal ions. The optimum growth time, synthesis temperature and C_2_H_2_ flow rate for the SS@CNTs synthesis were 45 min, 600 °C and 200 sccm, respectively, and the CNTs showed a larger length–diameter ratio, a higher specific surface area and better morphology compared to the CNTs synthesized under other conditions. The SS@CNTs adsorbent did well in the removal of Pb(II) and Cu(II) with maximum equilibrium adsorption capacities of 427.26 mg·g^−1^ and 132.79 mg·g^−1^, respectively, at 25 °C for an adsorption time of 90 min. The pseudo-second-order model and isotherms of the Langmuir model provided the most accurate fitting with the adsorption process of Pb(II) and Cu(II). The adsorption thermodynamics results indicated that the adsorption process was spontaneous and endothermic. SS@CNTs had a large specific surface area and abundant adsorption sites and types, owing to the combination of the advantages of CNTs and steel slag. In comparison to other adsorbent materials, SS@CNTs had a better adsorption performance for Pb(II) and Cu(II). In summary, SS@CNTs that utilize a steel industrial byproduct for raw material have good application prospects in the elimination of heavy metals, such as lead and copper, from wastewater.

## Figures and Tables

**Table 1:** Chemical composition of the steel slag.

Material	Chemical Composition/%								
	CaO	Fe_2_O_3_	SiO_2_	MgO	Al_2_O_3_	Na_2_O	f-CaO ^1^	LOI ^2^	Others
Steel slag	38.72	33.98	9.08	5.87	2.91	0.22	3.78	3.23	2.21

^1^ f-CaO: free calcium oxide; ^2^ LOI: loss on ignition.

**Table 2:** The corresponding parameters of TGA of the SS@CNTs synthesized using different time intervals.

Time (min)	T_0_ (°C)	T_f_ (°C)	Yield of Carbon (wt%)
15	461	640	13
30	465	657	23
45	463	672	36
60	463	673	39

**Table 3:** The BET surface areas and porosity characters of SS@CNTs synthesized using different time intervals.

Samples	S_BET_ (m^2^·g^−1^)	V_t_ (cm^3^·g^−1^)	D_A_ (nm)
Steel slag	2.05	0.0041	6.78
SS@CNTs-15	36.75	0.0404	3.9
SS@CNTs-30	43.19	0.061	4.48
SS@CNTs-45	49.85	0.0639	5.57
SS@CNTs-60	43.07	0.0635	5.9

**Table 4:** The corresponding parameters of TGA of the SS@CNTs synthesized at various temperatures.

Temperature (°C)	T_0_ (°C)	T_f_ (°C)	Yield of Carbon (wt%)
500	445	629	4.5
600	463	672	36
700	488	675	17
800	489	685	15

**Table 5:** BET surface areas and porosity characters of SS@CNTs synthesized at various temperatures.

Samples	S_BET_ (m^2^·g^−1^)	V_t_ (cm^3^·g^−1^)	D_A_ (nm)
SS@CNT-500	15.89	0.0247	5.5
SS@CNT-600	49.85	0.0639	5.57
SS@CNT-700	15.74	0.0364	8.26
SS@CNT-800	11.07	0.0195	6.5

**Table 6:** The parameters of TGA of the SS@CNTs synthesized under various C_2_H_2_ flow rates.

C_2_H_2_:N_2_	T_0_ (°C)	T_f_ (°C)	Yield of Carbon (wt%)
100:800	461	659	17
200:800	463	672	36
300:800	463	670	38

**Table 7:** The parameters of the TGA of the CNTs synthesized under different C_2_H_2_ flow rates.

Samples	S_BET_ (m^2^·g^−1^)	V_t_ (cm^3^·g^−1^)	D_A_ (nm)
SS@CNT-100	25.63	0.058	7.89
SS@CNT-200	49.85	0.061	4.48
SS@CNT-300	37.92	0.0727	14.63

**Table 8:** The parameters of the pseudo-first-order and pseudo-second-order models.

Heavy Metal	*q_e_*_,exp_ (mg·g^−1^)	Pseudo-First-Order			Pseudo-Second-Order		
		*q_e,_*_cal_ (mg·g^−1^)	*k_a_*	*R* ^2^	*q_e_*_,cal_ (mg·g^−1^)	*k_b_*	*R* ^2^
Pb(II)	427.26	186.64	0.0423	0.876	432.9	0.0008	0.980
Cu(II)	132.79	83.22	0.0298	0.912	137.93	0.0009	0.99

**Table 9:** The corresponding parameters of the adsorption isotherm fitting.

Heavy Metal	Temperature	Langmuir Model			Freundlich Model		
		*q_m_*	*K_L_*	*R* ^2^	*K_F_*	*n*	*R* ^2^
Pb(II)	288 K	492.61	0.238	0.9982	250.88	6.588	0.9571
	298 K	515.46	0.348	0.9989	287.88	7.297	0.9312
	318 K	537.63	0.598	0.9961	332.23	8.178	0.9175
Cu(II)	288 K	163.13	0.0367	0.9953	28.24	3.047	0.9644
	298 K	172.41	0.0448	0.9953	36.62	3.419	0.9481
	318 K	185.53	0.063	0.9962	50.34	3.949	0.9671

**Table 10:** The thermodynamics parameters of the adsorption process at different temperatures.

Heavy Metal	*T* (K)	Δ*H*^0^ (kJ·mol^−1^)	Δ*S*^0^ (J·mol^−1^·K^−1^)	Δ*G*^0^ (kJ·mol^−1^)	*R* ^2^
Pb(II)	288	23.15	112.95	−9.334	0.9921
	298			−10.597	
	318			−12.742	
Cu(II)	288	13.68	54.58	−2.029	0.9997
	298			−2.59	
	318			−3.669	

## Data Availability

Not applicable.

## References

[1] Al-Rashdi B.A.M., Johnson D.J., Hilal N. (2013). Removal of heavy metal ions by nanofiltration. Desalination.

[2] Stafiej A., Pyrzynska K. (2007). Adsorption of heavy metal ions with carbon nanotubes. Sep. Purif. Technol..

[3] Yu G., Lu Y., Guo J., Patel M., Bafana A., Wang X., Qiu B., Jeffryes C., Wei S., Guo Z. (2017). Carbon nanotubes, graphene, and their derivatives for heavy metal removal. Adv. Compos. Hybrid Mater..

[4] Jarup L. (2003). Hazards of heavy metal contamination. Br. Med. Bull..

[5] Qu X., Alvarez P.J., Li Q. (2013). Applications of nanotechnology in water and wastewater treatment. Water Res..

[6] Awual M.R., Ismael M., Yaita T., El-Safty S.A., Shiwaku H., Okamoto Y., Suzuki S. (2013). Trace copper(II) ions detection and removal from water using novel ligand modified composite adsorbent. Chem. Eng. J..

[7] Ni B.J., Huang Q.S., Wang C., Ni T.Y., Sun J., Wei W. (2019). Competitive adsorption of heavy metals in aqueous solution onto biochar derived from anaerobically digested sludge. Chemosphere.

[8] Ramos V.C., Utrilla J.R., Sanchez A.R., Ramon M.V.L., Polo M.S. (2021). Marble Waste Sludges as Effective Nanomaterials for Cu (II) Adsorption in Aqueous Media. Nanomaterials.

[9] Bezzina J.P., Ruder L.R., Dawson R., Ogden M.D. (2019). Ion exchange removal of Cu(II), Fe(II), Pb(II) and Zn(II) from acid extracted sewage sludge—Resin screening in weak acid media. Water Res..

[10] Ates N., Uzal N. (2018). Removal of heavy metals from aluminum anodic oxidation wastewaters by membrane filtration. Environ. Sci. Pollut. Res. Int..

[11] Chen Q., Yao Y., Li X., Lu J., Zhou J., Huang Z. (2018). Comparison of heavy metal removals from aqueous solutions by chemical precipitation and characteristics of precipitates. J. Water Process Eng..

[12] Fiyadh S.S., AlSaadi M.A., Jaafar W.Z., AlOmar M.K., Fayaed S.S., Mohd N.S., Hin L.S., El-Shafie A. (2019). Review on heavy metal adsorption processes by carbon nanotubes. J. Clean. Prod..

[13] Ouni L., Ramazani A., Taghavi Fardood S. (2019). An overview of carbon nanotubes role in heavy metals removal from wastewater. Front. Chem. Sci. Eng..

[14] Abdullah N., Yusof N., Lau W.J., Jaafar J., Ismail A.F. (2019). Recent trends of heavy metal removal from water/wastewater by membrane technologies. J. Ind. Eng. Chem..

[15] Salam M.A., Al-Zhrani G., Kosa S.A. (2012). Simultaneous removal of copper(II), lead(II), zinc(II) and cadmium(II) from aqueous solutions by multi-walled carbon nanotubes. Comptes Rendus Chim..

[16] Al-Sareji O.J., Abdulredha M., Mubarak H.A., Grmasha R.A., Alnowaishry A., Kot P., Al-Khaddar R., AlKhayyat A. (2021). Copper removal from water using carbonized sawdust. IOP Conf. Ser. Mater. Sci. Eng..

[17] Jellali S., Azzaz A.A., Jeguirim M., Hamdi H., Mlayah A. (2021). Use of lignite as a low-cost material for cadmium and copper removal from aqueous solutions: Assessment of adsorption characteristics and exploration of involved mechanisms. Water.

[18] Ihsanullah, Abbas A., Al-Amer A.M., Laoui T., Al-Marri M.J., Nasser M.S., Khraisheh M., Atieh M.A. (2016). Heavy metal removal from aqueous solution by advanced carbon nanotubes: Critical review of adsorption applications. Sep. Purif. Technol..

[19] Li Y.-H., Ding J., Luan Z., Di Z., Zhu Y., Xu C., Wu D., Wei B. (2003). Competitive adsorption of Pb2+, Cu2+ and Cd2+ ions from aqueous solutions by multiwalled carbon nanotubes. Carbon.

[20] Li H., Wei C., Zhang D., Pan B. (2019). Adsorption of bisphenol A on dispersed carbon nanotubes: Role of different dispersing agents. Sci. Total Environ..

[21] Rodriguez C., Leiva E. (2019). Enhanced Heavy Metal Removal from Acid Mine Drainage Wastewater Using Double-Oxidized Multiwalled Carbon Nanotubes. Molecules.

[22] Oliveira A.R., Correia A.A., Rasteiro M.G. (2021). Heavy Metals Removal from Aqueous Solutions by Multiwall Carbon Nanotubes: Effect of MWCNTs Dispersion. Nanomaterials.

[23] Wang B., Li F., Yang P., Yang Y., Hu J., Wei J., Yu Q. (2018). In Situ Synthesis of Diatomite–Carbon Nanotube Composite Adsorbent and Its Adsorption Characteristics for Phenolic Compounds. J. Chem. Eng. Data.

[24] Yang L., Wen T., Wang L., Miki T., Bai H., Lu X., Yu H., Nagasaka T. (2019). The stability of the compounds formed in the process of removal Pb(II), Cu(II) and Cd(II) by steelmaking slag in an acidic aqueous solution. J. Environ. Manage..

[25] Shi C., Wang X., Zhou S., Zuo X., Wang C. (2022). Mechanism, application, influencing factors and environmental benefit assessment of steel slag in removing pollutants from water: A review. J. Water Process Eng..

[26] Bing L., Biao T., Zhen M., Hanchi C., Hongbo L. (2019). Physical and Chemical Properties of Steel Slag and Utilization Technology of Steel Slag at Home and Abroad. IOP Conf. Ser. Earth Environ. Sci..

[27] Azira A.A., Suriani A.B., Rusop M. (2011). Carbon nanotubes formation from Fe/Ni/Mg by camphor oil decomposition. J. Ceram. Soc. Jpn..

[28] Nasibulin A.G., Koltsova T., Nasibulina L.I., Anoshkin I.V., Semencha A., Tolochko O.V., Kauppinen E.I. (2013). A novel approach to composite preparation by direct synthesis of carbon nanomaterial on matrix or filler particles. Acta Mater..

[29] Wang K., Qian C., Wang R. (2016). The properties and mechanism of microbial mineralized steel slag bricks. Constr. Build. Mater..

[30] See C.H., Dunens O.M., MacKenzie K.J., Harris A.T. (2008). Process Parameter Interaction Effects during Carbon Nanotube Synthesis in Fluidized Beds. Ind. Eng. Chem. Res..

[31] Chekin F., Raoof J.-B., Bagheri S., Hamid S.B.A. (2012). Fabrication of Chitosan-Multiwall Carbon Nanotube Nanocomposite Containing Ferri/Ferrocyanide: Application for Simultaneous Detection ofD-Penicillamine and Tryptophan. J. Chin. Chem. Soc..

[32] Soltani R., Sani M.A.F., Mohaghegh F. (2014). The Effect of Temperature and N2:C2H2Flow Rate on the Growth of Carbon Nanotubes Synthesized by CCVD of Acetylene on Alumina–Zirconia Matrix. Fuller. Nanotub. Carbon Nanostructures.

[33] Allaedini G., Tasirin S.M., Aminayi P. (2015). Synthesis of CNTs via chemical vapor deposition of carbon dioxide as a carbon source in the presence of NiMgO. J. Alloy. Compd..

[34] Yang H., Cahela D.R., Tatarchuk B.J. (2008). A study of kinetic effects due to using microfibrous entrapped zinc oxide sorbents for hydrogen sulfide removal. Chem. Eng. Sci..

[35] Chang B.-K., Lu Y., Tatarchuk B.J. (2006). Microfibrous entrapment of small catalyst or sorbent particulates for high contacting-efficiency removal of trace contaminants including CO and H2S from practical reformates for PEM H2–O2 fuel cells. Chem. Eng. J..

[36] Qin J., Wang C., Yao Z., Ma Z., Cui X., Gao Q., Wang Y., Wang Q., Wei H. (2021). Influencing factors and growth kinetics analysis of carbon nanotube growth on the surface of continuous fibers. Nanotechnology.

[37] Ducati C., Alexandrou I., Chhowalla M., Amaratunga G.A.J., Robertson J. (2002). Temperature selective growth of carbon nanotubes by chemical vapor deposition. J. Appl. Phys..

[38] Chaisitsak S., Nukeaw J., Tuantranont A. (2007). Parametric study of atmospheric-pressure single-walled carbon nanotubes growth by ferrocene–ethanol mist CVD. Diam. Relat. Mater..

[39] Tripathi N., Mishra P., Harsh H., Islam S.S. (2014). Fine-tuning control on CNT diameter distribution, length and density using thermal CVD growth at atmospheric pressure: An in-depth analysis on the role of flow rate and flow duration of acetylene (C2H2) gas. Appl. Nanosci..

[40] Tofighy M.A., Mohammadi T. (2011). Adsorption of divalent heavy metal ions from water using carbon nanotube sheets. J. Hazard. Mater..

[41] Lasheen M.R., El-Sherif I.Y., Sabry D.Y., El-Wakeel S.T., El-Shahat M.F. (2013). Removal of heavy metals from aqueous solution by multiwalled carbon nanotubes: Equilibrium, isotherms, and kinetics. Desalination Water Treat..

[42] Dehghani M.H., Taher M.M., Bajpai A.K., Heibati B., Tyagi I., Asif M., Agarwal S., Gupta V.K. (2015). Removal of noxious Cr (VI) ions using single-walled carbon nanotubes and multi-walled carbon nanotubes. Chem. Eng. J..

[43] Xu P., Zeng G.M., Huang D.L., Lai C., Zhao M.H., Wei Z., Li N.J., Huang C., Xie G.X. (2012). Adsorption of Pb(II) by iron oxide nanoparticles immobilized Phanerochaete chrysosporium: Equilibrium, kinetic, thermodynamic and mechanisms analysis. Chem. Eng. J..

[44] Rodrigues E., Almeida O., Brasil H., Moraes D., Reis M.A.L.d. (2019). Adsorption of chromium (VI) on hydrotalcite-hydroxyapatite material doped with carbon nanotubes: Equilibrium, kinetic and thermodynamic study. Appl. Clay Sci..

[45] Muthukumaran C., Sivakumar V.M., Thirumarimurugan M. (2016). Adsorption isotherms and kinetic studies of crystal violet dye removal from aqueous solution using surfactant modified magnetic nanoadsorbent. J. Taiwan Inst. Chem. Eng..

[46] Cristiano E., Hu Y.-J., Siegfried M., Kaplan D., Nitsche H. (2011). A Comparison of Point of Zero Charge Measurement Methodology. Clays Clay Miner..

[47] Huifen Y., Wen M., Weina Z., Zhiyong W. Steel Slag as Multi-functional Material for Removal of Heavy Metal Ions in Wastewater. Proceedings of the 2011 International Conference on Computer Distributed Control and Intelligent Environmental Monitoring.

[48] Lim J.W., Chew L.H., Choong T.S.Y., Tezara C., Yazdi M.H. (2016). Utilizing steel slag in environmental application—An overview. IOP Conf. Ser. Earth Environ. Sci..

[49] Ortiz N., Pires M.A.F., Bressiani J.C. (2001). Use of steel converter slag as nickel adsorber to wastewater treatment. Waste Manag..

[50] Duan C., Ma T., Wang J., Zhou Y. (2020). Removal of heavy metals from aqueous solution using carbon-based adsorbents: A review. J. Water Process Eng..

[51] Bassyouni M., Mansi A.E., Elgabry A., Ibrahim B.A., Kassem O.A., Alhebeshy R. (2019). Utilization of carbon nanotubes in removal of heavy metals from wastewater: A review of the CNTs’ potential and current challenges. Appl. Phys. A.

[52] Farghali A.A., Abdel Tawab H.A., Abdel Moaty S.A., Khaled R. (2017). Functionalization of acidified multi-walled carbon nanotubes for removal of heavy metals in aqueous solutions. J. Nanostructure Chem..

[53] Ma Y.-X., Shao W.-J., Sun W., Kou Y.-L., Li X., Yang H.-P. (2018). One-step fabrication of β-cyclodextrin modified magnetic graphene oxide nanohybrids for adsorption of Pb(II), Cu(II) and methylene blue in aqueous solutions. Appl. Surf. Sci..

[54] Qu G., Zhou J., Liang S., Li Y., Ning P., Pan K., Ji W., Tang H. (2022). Thiol-functionalized multi-walled carbon nanotubes for effective removal of Pb(II) from aqueous solutions. Mater. Chem. Phys..

[55] Nicola R., Costişor O., Ciopec M., Negrea A., Lazău R., Ianăşi C., Picioruş E.-M., Len A., Almásy L., Szerb E.I. (2020). Silica-Coated Magnetic Nanocomposites for Pb2+ Removal from Aqueous Solution. Appl. Sci..

[56] Manyangadze M., Chikuruwo N.M.H., Narsaiah T.B., Chakra C.S., Charis G., Danha G., Mamvura T.A. (2020). Adsorption of lead ions from wastewater using nano silica spheres synthesized on calcium carbonate templates. Heliyon.

[57] Xin S., Zeng Z., Zhou X., Luo W., Shi X., Wang Q., Deng H., Du Y. (2017). Recyclable Saccharomyces cerevisiae loaded nanofibrous mats with sandwich structure constructing via bio-electrospraying for heavy metal removal. J. Hazard. Mater..

[58] Tofighy M.A., Mohammadi T. (2016). Copper ions removal from aqueous solutions using acid-chitosan functionalized carbon nanotubes sheets. Desalination Water Treat..

[59] Lei D.-y., Liu Z., Peng Y.-h., Liao S.-b., Xu H. (2013). Biosorption of copper, lead and nickel on immobilized Bacillus coagulans using experimental design methodologies. Ann. Microbiol..

